# Socio-economic inequalities in social network, loneliness and mental health during the COVID-19 pandemic

**DOI:** 10.1177/0020764020976694

**Published:** 2020-12-07

**Authors:** Rusi Jaspal, Glynis M Breakwell

**Affiliations:** 1 Nottingham Trent University, Nottingham, UK; 2 Imperial College, London, UK; 3 University of Bath, Bath, UK

**Keywords:** Health inequalities, neighbourhood identification, perceived risk of COVID, mistrust, depression, anxiety

## Abstract

**Background:**

During the COVID-19 pandemic, a focus on isolation and loneliness is important, especially as social distancing policies (which for some groups involve self-isolation or quarantine) are likely to accentuate these experiences and affect mental health.

**Aims:**

This study focuses on socio-economic inequalities in social network, loneliness and mental health during the COVID-19 pandemic.

**Methods:**

Two-hundred and fourteen residents of Wandsworth, a South West London Borough in the United Kingdom completed an online cross-sectional survey on the impact of COVID-19 on mental health. Data were analysed using independent samples *t*-tests and multiple regression.

**Results:**

Middle-aged people reported a less strong social network and more loneliness, anxiety and depression than younger people. People with a long-term health condition reported a less strong social network, more loneliness, more general practitioner (GP) and hospital visits, and poorer mental health than those with no long-term health conditions. People receiving State financial benefits reported less use of public spaces, a less strong social network, more loneliness, more GP and hospital visits and poorer mental health than those not receiving benefits. Greater neighbourhood identification was associated with a stronger social network and better mental health outcomes. Multiple regression analyses showed that, over and above loneliness, perceived personal risk of COVID-19 constitutes an additional precipitant for both depression and anxiety when controlling for other variables.

**Conclusion:**

As a novel stressor associated with the pandemic, the situational and involuntary perception of being at risk of COVID-19 may be stimulating anxiety and depressive symptomatology, which will need to be managed effectively as resurgences of the disease are predicted and communicated to the general public under growing mistrust and uncertainty.

## Introduction

The deleterious effects of social isolation and loneliness on mental and physical health outcomes have long been recognised. There is now considerable evidence that older people, in particular, are at higher risk of social isolation, loneliness and, thus, poor mental and physical health outcomes (Erzen & Çikrikci, 2018; Golden et al., 2009; Singh & Misra, 2009). This has meant that there has been a recent focus on the health and wellbeing of older people in interventions, campaigns and initiatives introduced by local government authorities in the United Kingdom (UK) (e.g. Wandsworth Borough Council, 2015). However, there has been less insight into the relationship between social isolation, loneliness and mental health in other potentially vulnerable communities, particularly those with lower income, people receiving State financial benefits and those with long-term health conditions. These are people who may lack the necessary social and economic capital to cope effectively with emergent social psychological ‘stressors’, such as the outbreak of COVID-19. In view of the pandemic, renewed focus on these social psychological challenges is necessary, especially as social distancing policies (which for some groups involve self-isolation or quarantine) are likely to accentuate feelings of isolation and loneliness (Jetten et al., 2020). Social psychological factors, such as neighbourhood identification, participation in and engagement with one’s local community (such as volunteering and use of public spaces), prior loneliness, strength of social network and perceived personal risk of COVID-19 are likely to determine, at least in part, people’s mental health during the unprecedented circumstances brought about by the dangers of COVID-19. In this article, the role of socio-economic and health inequalities in strength of social network, loneliness, perceived personal risk of COVID-19 and mental health (depression and anxiety) is examined in a sample of adults in the London borough of Wandsworth.

### Social network, neighbourhood identification and loneliness

There is evidence that both strength of social network and loneliness play a significant causal role in the onset of depression and anxiety in the population as a whole (Erzen & Çikrikci, 2018) and that older people, in particular, are at disproportionately high risk (Golden et al., 2009; Singh & Misra, 2009). In their study of Spanish people aged 50 and above, Domènech-Abella et al. (2017) found that the type and size of one’s social network mediated the relationship between loneliness and depression. Similarly, in an Irish study, Domènech-Abella et al. (2019) provide evidence that both objective factors, such as the size of one’s social network, and subjective factors, such as loneliness, should be concurrently examined in addressing anxiety in older people. In research into younger adults, loneliness (as opposed to strength of social network) emerges as the most robust predictor of depression (Matthews et al., 2016; see also Vasileiou et al., 2019).

Alpass and Neville (2003) found that social isolation (i.e. having a weak social network) had an impact on depression in older men, which they attributed to factors such as the loss of a professional identity, physical mobility issues and the loss of valued relationships. It has also been found that the availability of social support mediates the relationship between loneliness and depression (Liu et al., 2016). Social support can be derived in many different ways and includes engagement with one’s local community though volunteering and use of communal spaces (such as local charities, community centres and parks) (Pilkington et al., 2012). The likelihood of engaging with one’s local community in this way is likely to be tied to neighbourhood identification.

The social cure perspective in social psychology (Jetten et al., 2012) posits that identification with relevant and meaningful social groups, such as one’s neighbourhood, performs positive functions for both psychological and physical support primarily by providing a sense of inclusion, belonging and resilience in the face of stressors. Neighbourhood identification refers to the extent to which people see their neighbourhood as an element of their identity – it can be assessed in terms of the level of importance one appends to one’s neighbourhood, how happy one feels about being a resident of it, how fulfilled one feels by it, and the extent to which one’s neighbourhood affiliation guides one’s behaviour and self-presentation.

Neighbourhood identification has also been found to be related to mental health outcomes. Fong et al. (2019a) showed that higher neighbourhood identification was directly associated with better mental health and attenuated the effects of living in a neighbourhood with low socio-economic status on mental health. Fong et al. (2019b) also showed that neighbourhood identification may perform a protective role against poor mental health associated with negative change in one’s local environment. It can also be hypothesised that this form of social identification may also be protective against the stressors associated with COVID-19. Indeed, emerging research has found that perceived risk of COVID-19 is associated with negative affect, such as fear, as well as imposed identity change (Breakwell & Jaspal, in press; Harper et al., 2020), suggesting that perceived personal risk constitutes a significant stressor during the pandemic. Since COVID-19 induces existential fear (the severity of damage), exacerbates uncertainty and insecurity (as an ‘invisible enemy’), and incites interpersonal mistrust (anyone can infect you), it can be hypothesised that perceived personal risk will also be associated with depression and anxiety.

Stafford et al. (2011) have shown that neighbourhood cohesion (as an aspect of neighbourhood identity) was inversely associated with depressive symptomatology independent of demographic and socio-economic factors, suggesting that the ability to build friendships within one’s neighbourhood is associated with a lower risk of poor mental health. Unsurprisingly, the physical composition of one’s neighbourhood (such as the availability of good housing and healthcare, and safe communal spaces) is also likely to play a role in social and psychological outcomes, such as neighbourhood identification and indeed mental health (Stockton, 2014). Thus, neighbourhood identification must be viewed as part of a system of factors, including engaging with one’s community, that potentially determine mental health outcomes during the pandemic.

### Health and socio-economic inequalities

People from varying age groups and socio-economic backgrounds, and with different physical and mental health profiles may react differently to emergent stressors with the capacity to challenge mental health (Jaspal, Lopes, et al., 2020). It is important to examine the impact of this socio-economic diversity.

Evidence on the relationship between income inequality and mental health has appeared contradictory or inconclusive but this may be because researchers are not always measuring the same things and, if they are, they measure them in different ways. In their US study, Sturm and Gresenz (2002) found no evidence of an association between income inequality and depression. Conversely, Wildman (2003) has found that subjective financial status was a major determinant of ill health in a UK sample. Increasingly, it is recognised that social psychological factors are likely to mediate the potential relationship between income inequality and poor mental health outcomes. In their multilevel analysis of Welsh adults, Fone et al. (2007) found that income deprivation was associated with poor mental health and that social cohesion mediated this relationship. Zimmerman and Bell (2006) showed that social capital is also an important mediator. Being in receipt of benefits (that is, financial support from the government for the unemployed, those with ill health and those facing economic deprivation) is one indicator of income inequality. Being in receipt of benefits is also socially stigmatised and can reflect marginalisation (Garthwaite, 2015). In view of previous research, it is hypothesised that people who are receiving benefits will report a less strong social network, more loneliness and poorer mental health than those who are not receiving benefits.

There is also evidence that poor physical health is associated with social isolation, loneliness and poor mental health (Mushtaq et al., 2014). In fact, the effects of loneliness on mental health have been shown to be mediated by poor physical health (Swami et al., 2007). Yet, loneliness itself has been observed to increase the risk of poor physical health outcomes (Jaremka et al., 2013). There is a mutually reinforcing relationship between loneliness and physical illness. Accordingly, those with long-term health conditions may exhibit a less strong social network, more loneliness and decreased community participation (e.g. volunteering, use of public spaces). As likely indicators of physical health impairments, the number of visits to one’s general practitioner (GP) and to hospital in the past 12 months should be positively associated with social isolation, loneliness and poor mental health outcomes.

### Hypotheses

The following hypotheses are proposed and tested:

1. Older people will report a less strong social network, more loneliness, anxiety and depression than younger people.
2. People who have a long-term health condition will report a less strong social network, more loneliness, more GP and hospital visits, and poorer mental health than those with no long-term health conditions.
3. People who are receiving benefits will report less use of public spaces, a less strong social network, more loneliness, more GP and hospital and poorer mental health than those who are not receiving benefits.
4. Neighbourhood identification will be associated with a stronger social network and better mental health.
5. Loneliness and perceived personal risk of COVID-19 will emerge as the strongest predictors of poor mental health, when controlling for other variables.

## Method

### Ethics

This project received ethics approval from Nottingham Trent University’s College of Business, Law and Social Sciences Ethics Committee (2020/226). Participants provided electronic consent to participate, were debriefed and thanked for their time.

### Participants

Two-hundred and fourteen residents in Wandsworth completed an online survey on the impact of COVID-19 on psychological wellbeing. Wandsworth is a borough located in South West London with an estimated population of 323,357 in 2017 (Wandsworth Borough Council, 2019), and has one the lowest rates of income inequality, poverty and unemployment of all London boroughs (Trust for London, 2017). The borough has less than half the unemployment rate of both London and the country and is ranked seventh in the country for proportion of people employed in professional or technical occupations (Wandsworth Borough Council, 2019). The survey was publicised by Wandsworth Borough Council on social media and by local charities and organisations that work in collaboration with the Council. Participants were recruited in the first 3 weeks of September 2020. There were two sole eligibility criteria: (1) being aged 18 or over and (2) being a resident of the London borough of Wandsworth. See Table 1 for a full summary of the socio-demographic characteristics of the participant sample.

**Table 1.** Socio-demographic characteristics of the sample.

Age groups	Young adults (29–40)	Middle-aged (41–65)	Older adults (66–89)						
	31 (14.5%)	107 (50%)	76 (35.5%)						
Citizenship	British	European Union							
	*N* = 196 (91.6%)	*N* = 18 (8.4%)							
Ethnicity	White British	White other	White and Black Caribbean	White and Asian	Pakistani	Indian	Asian other	Caribbean	African
	*N* = 151 (70.6%)	*N* = 41 (19.2%)	*N* = 4 (1.9%)	*N* = 2 (0.9%)	*N* = 2 (0.9%)	*N* = 3 (1.4%)	*N* = 3 (1.4%)	*N* = 2 (0.9%)	*N* = 6 (2.8%)
Religion	No religion	Christianity	Islam	Hinduism	Judaism	Other			
	*N* = 92 (43%)	*N* = 107 (50%)	*N* = 5 (2.3%)	*N* = 3 (1.4%)	*N* = 2 (0.9%)	*N* = 5 (2.3%)			
Relationship Status	Single	Married	Unmarried – with partner	Divorced/separated	Civil partnership	Widowed			
	*N* = 91 (42.5%)	*N* = 60 (28%)	*N* = 14 (6.5%)	*N* = 36 (16.8%)	*N* = 3 (1.4%)	*N* = 10 (4.7%)			
Living arrangement	Alone	With partner	With family	With children	With unrelated housemates	With parents	Other		
	106 (49.5%)	55 (25.7%)	26 (12.1%)	13 (6.1%)	9 (4.2%)	3 (1.4%)	2 (0.9%)		
Income	Less than £10,000	£10,000 to £19,999	£20,000 to £29,999	£30,000 to £39,999	£40,000 to £49,999	£50,000 to £59,999	£60,000 or more		
	*N* = 33 (15.4%)	*N* = 40 (18.7%)	*N* = 37 (17.3%)	*N* = 37 (17.3%)	*N* = 18 (8.4%)	*N* = 6 (2.8%)	*N* = 43 (20.1%)		
Education	Undergraduate degree	A-/AS-Levels	GCSE/O Level	Postgraduate degree	Apprenticeship	Other	None		
	*N* = 61 (28.5%)	*N* = 30 (14%)	*N* = 26 (12.1%)	*N* = 79 (36.9%)	*N* = 1 (0.5%)	*N* = 1 (0.5%)	*N* = 16 (7.5%)		
Gender	Male	Female							
	51 (23.8%)	163 (76.2%)							
Long-term health condition	Yes119 (55.6%)	No95 (44.4%)							
Benefits	Yes	No							
	42 (19.6%)	172 (80.4%)							

### Measures

Participants were asked to indicate their age, gender, citizenship, ethnicity, religion, relationship status, living arrangement, level of education, employment status and income. They were also asked about long-term health conditions, how many times they had seen their GP and/or been to hospital in the last 12 months, and whether or not they were in receipt of benefits.

***Neighbourhood identification*** was measured using an adapted version of the positive affect and enactment items relating to identity (Vignoles et al., 2006). The scale consists of five items focusing specifically upon identification with Wandsworth where the study was conducted (e.g. ‘How important is being a Wandsworth resident in defining who you are?). The items were measured on a 5-point scale (1 = not at all important, 5 = extremely important). The scale manifested good reliability (α = .79).

***Loneliness*** was measured using the 6-Item (Short) De Jong Gierveld Loneliness Scale (De Jong Gierveld & Van Tilburg, 2006). The scale consists of six items (e.g. ‘I miss having people around’), which were measured on a 5-point scale (1 = none of the time, 5 = all of the time). The scale manifested excellent reliability (α = .88).

***Strength of social network*** was measured using the Lubben Social Network Scale (Lubben et al., 2006), which consists of six items (e.g. ‘How many relatives do you see or hear from at least once a month?). Items were measured on a 7-point scale (0 = none, 6 = 9 or more). The scale manifested excellent reliability (α = .89).

***Perceived personal risk of COVID-19*** was measured using the COVID-19 Own Risk Appraisal Scale (CORAS) (Jaspal, Fino, et al., 2020), which consists of six items (e.g. ‘I am sure I will NOT get infected with COVID-19.’) Items were measured a 5-point scale (1 = strongly disagree, 5 = strongly agree). The scale manifested excellent reliability (α = .84).

***Depression*** was measured using the Center for Epidemiologic Studies Depression Scale Revised (Radloff, 1977), which consists of 10 items (e.g. ‘I felt hopeful about the future.’) Items were measured on a 4-point scale (0 = rarely or none of the time, 3 = all of the time). The scale manifested excellent reliability (α = .92).

***Anxiety*** was measured using the Generalized Anxiety Disorder Assessment (Spitzer et al., 2006), which consists of seven items (e.g. ‘Worrying too much about different things?’). Items were measured on a 4-point scale (0 = not at all, 3 = nearly every day). The scale manifested excellent reliability (α = .92)

***Volunteering, exercise and use of public spaces*** were measured using two items for each of the three behaviours. First, participants were asked how many days per week they engaged in these three behaviours and, second, they were asked to indicate the number of minutes for each session. The number of days and number of minutes were multiplied, providing three separate scores for the amount of volunteering; exercise; and use of public spaces (e.g. libraries, parks, community centres), respectively.

## Results

### Descriptive statistics

Please see Table 2 for a full summary of the descriptive statistics for the key variables of this study.

**Table 2.** Descriptive statistics for the key variables of this study.

Continuous variables	Mean	*SD*	Minimum	Maximum
Number of GP visits	2.74	3.23	0	20
Number of hospital visits	1.72	3.39	0	30
Neighbourhood identification	14.14	3.75	5	24
Loneliness	16.83	5.16	6	29
Perceived risk of COVID-19	19.57	4.34	7	30
Use of public spaces	125.15	136.41	0	840
Exercise	151.56	147.33	0	840
Volunteering	103.86	370.68	0	3,360
Depression	12.13	7.74	0	29
Anxiety	6.51	5.59	0	21

### Differences between age groups

A multivariate one-way ANOVA bootstrapped at 1000 samples showed statistically significant main effects of age group (younger adults [29–40 years], middle-aged adults [41–65 years] vs older adults [66–89 years]) on loneliness [*F*(2,213) = 5.049, *p* = .007], strength of social network [*F*(2,213) = 4.445, *p* = .013], depression [*F*(2,213) = 6.308, *p* = .002] and anxiety [*F*(2,213) = 7.568, *p* = .001].

Post-hoc LSD tests showed that middle-aged adults (*M* = 17.72, *SD* = 5.04) reported higher loneliness than older adults (*M* = 15.36, SD = 4.67, *p* = .002, 95% confidence intervals [CIs] .8681, 3.8606); that middle-aged adults reported a less strong social network (*M* = 14.22, *SD* = 8.13) than older adults (*M* = 17.86, *SD* = 8.15, *p* = .003, 95% CIs –6.0503, –1.2116); that older adults reported less depression (*M* = 9.66, *SD* = 6.83) than both younger adults (*M* = 13.32, *SD* = 8.11, *p* = .024, 95% CIs –6.8363, –.4931) and middle-aged adults (*M* = 13.54, *SD* = 7.86, *p* = .001, 95% CIs –6.1074, –1.6423); and that older adults reported less anxiety (*M* = 4.59, *SD* = 4.73) than both younger adults (*M* = 7.00, *SD* = 5.07, *p* = .039, 95% CIs –4.6883, -.1275) and middle-aged adults (*M* = 7.73, *SD* = 5.96, *p*<.001 95% CIs –4.7421, –1.5316).

The results suggest that middle-aged people report the least strong social network and the most loneliness, depression and anxiety of all age groups and, thus, only partially support hypothesis 1.

### Interaction between long-term health condition groups (yes vs no) and benefits groups (yes vs no)

A chi-squared test showed that people with long-term health conditions were no more likely than those with no long-term health conditions to be receiving benefits (*p*>0.05). Thus, having a long-term health condition and being in receipt of benefits were regarded as separate categories and independent samples *t*-tests were conducted to examine differences between the groups within them.

### Differences between people with health long-term conditions vs those with no long-term health conditions

Independent samples *t*-tests bootstrapped at 1000 samples showed that those with long-term health conditions reported a less strong social network [*t*(212) = 2.553, *p* = .016], more loneliness [*t*(212) = –2.224, *p* = .024], more GP and hospital visits [*t*(212) = –3.833, *p* = .001 and *t*(212) = –4.881, *p* = .001, respectively] and higher depression [*t*(212) = –2.838, *p* = .004] than those with no long-term health conditions. Please see Table 3 for the means, standard deviations, power analyses and CIs for the independent samples *t*-tests. The findings support hypothesis 2.

**Table 3.** Independent samples *t*-tests for people with a long-term health condition versus those with no long-term health condition for key variables of interest.

	Long-term health condition		No long-term health condition		*p-*value (two-tailed)	Cohen’s *D*	95% confidence intervals
	*N* = 119		*N* = 95				
Strength of social network	*M*	*SD*	*M*	*SD*	.022	0.35	.62106, 5.17406
	14.54	7.82	17.42	8.67			
Loneliness	*M*	*SD*	*M*	*SD*	.020	0.31	–2.92918, –.19523
	17.52	5.09	15.96	5.13			
Number of GP visits	*M*	*SD*	*M*	*SD*	.002	0.53	–2.55065, –.77924
	3.47	3.45	1.82	2.67			
Number of hospital visits	*M*	*SD*	*M*	*SD*	.001	0.70	–3.03570, –1.39958
	2.68	4.1	0.52	1.54			
Depression	*M*	*SD*	*M*	*SD*	.004	0.39	–4.86513, –.99760
	13.45	7.87	10.47	7.27			

### Differences between people receiving benefits vs those not receiving benefits

Further independent samples *t*-tests bootstrapped at 1000 samples showed that those receiving benefits reported less use of public spaces [*t*(212) = 2.115, *p* = .006], more loneliness [*t*(212) = 2.580, *p* = .012], a less strong social network [*t*(212) = 2.115, *p* = .020], more GP and hospital visits [*t*(212) = –3.794, *p* = .005 and *t*(212) = –3.420, *p* = .032, respectively], higher depression [*t*(212) = 2.679, *p* = .008] and higher anxiety [*t*(212) = –2.034, *p* = .039] than those who were not receiving benefits. Please see Table 4 for the means, standard deviations, power analyses and CIs for the independent samples *t*-tests. These findings support hypothesis 3.

**Table 4.** Independent samples *t*-tests for people receiving benefits versus those who are not receiving benefits for key variables of interest.

	Receiving benefits		Not receiving benefits		*p-*value (two-tailed)	Cohen’s *D*	95% confidence intervals
	*N* = 42		*N* = 172				
Use of public spaces	*M*	*SD*	*M*	*SD*	.014	0.46	11.92578, 103.37959
	78.81	109.67	136.46	140.13			
Strength of social network	*M*	*SD*	*M*	*SD*	.036	0.37	.20471, 5.79972
	13.40	7.86	16.41	8.33			
Loneliness	*M*	*SD*	*M*	*SD*	.011	0.44	–3.98539, –.53288
	18.64	5.21	16.38	5.06			
Number of GP visits	*M*	*SD*	*M*	*SD*	<.001	0.56	–3.10552, –.98196
	4.38	4.41	2.34	2.73			
Number of hospital visits	*M*	*SD*	*M*	*SD*	.001	0.46	–3.07158, –.82543
	3.29	5.38	1.34	2.58			
Depression	*M*	*SD*	*M*	*SD*	.008	0.46	–6.10356, –.92911
	14.95	7.72	11.44	7.60			
Anxiety	*M*	*SD*	*M*	*SD*	.043	0.35	–3.82734, –.05970
	8.07	5.42	6.13	5.58			

### Correlations

Income was associated with more use of public spaces, more exercise and strength of social network but negatively associated with loneliness. Frequency of GP visits was positively associated with loneliness, perceived risk of COVID-19, depression and anxiety. Frequency of hospital visits was positively associated with depression. Neighbourhood identification was positively associated with strength of social network and exercise but negatively associated with loneliness and depression. Strength of social network was negatively associated with loneliness, anxiety and depression and positively associated with exercise and use of public spaces. Loneliness was negatively associated with exercise and positively associated with perceived risk of COVID-19, depression and anxiety. Perceived risk of COVID-19 was positively associated with both depression and anxiety. Exercise was negatively associated with depression and anxiety. Use of public spaces was negatively associated with depression. Anxiety was positively associated with depression. See Table 5 for a full summary of the correlations. These findings support hypothesis 4.

**Table 5.** Correlations between the main variables of interest.

	1	2	3	4	5	6	7	8	9	10	11	12
1.Income		–.13	–.14*	.06	.21**	–.16*	.04	.21**	.17*	.05	–.12	–.08
2.Number of GP visits	–.13		.29**	–.10	–.07	.13*	.15*	.01	–.10	–.06	.19**	.17*
3.Number of hospital visits	–.14*	.29**		.00	–.07	.10	.07	.01	–.04	–.08	.15*	.09
4.Neighbourhood identification	.06	–.10	.00		.22**	–.19*	.04	.08	.18*	.12	–.22**	–.13*
5.Strength of social network	.21**	–.06	–.07	.22**		–.65*	–.08	.24**	.31**	.06	–.48**	–.39**
6.Loneliness	–.16*	.14*	.10	–.19**	–.65**	.19*	.19**	–.12	–.32**	–.06	.77**	.65*
7.Perceived risk of COVID –19	.04	.15*	.07	.04	–.08			.02	.04	–.03	.28**	.30**
8.Use of public spaces	.21**	.01	.01	.08	.24**	–.12	.02		.45**	–.00	–.14*	–.05
9.Exercise	.17*	–.10	–.04	.18**	.31**	–.32**	.04	.45**		–.06	–34**	–.25**
10.Volunteering	.05	–.06	.08	.12	.059	–.06	–.03	–.00	–.06		–.08	–.13
11.Depression	–.12	.19**	.15*	–.22**	–.48**	.77**	.28**	–.14*	–.34**	–.08		.83**
12.Anxiety	–.08	.17*	.09	–.13	–.39**	.65**	.30**	–.05	–.25**	–.13	.83**	

* *p* < .05; ** *p* < .001.

### Multiple regression models

Please see Table 6 for a full summary of the multiple regression models predicting depression and anxiety.

**Table 6.** Stepwise regression models predicting the variance of depression and anxiety, respectively.

Depression	Model 1				Model 2				Model 3			
Predictors	*B*	*SE*	β	*t*	*B*	*SE*	β	*t*	*B*	*SE*	β	*t*
Loneliness	1.15	.07	.77**	17.34	1.10	.07	.73**	15.85	1.05	.07	.70**	15.12
Exercise					–.01	.00	–.10*	–2.18	–.01	.00	–.12*	–2.58
Perceived risk of COVID-19									.26	.08	.15**	3.38
*R* ^2^	.58				.59				.61			
*F* change	300.63**				4.74*				11.44**			
Anxiety	Model 1				Model 2				Model 3			
Predictors	*B*	*SE*	β	*t*	*B*	*SE*	β	*t*	*B*	*SE*	β	*t*
Loneliness	.71	.06	.65**	12.53	.68	.06	.63**	11.88	.65	.06	.60**	11.38
Older adulthood					–1.42	.62	–.12*	–2.31	–1.30	.60	–.12*	–2.17
Perceived risk of COVID-19									.22	.07	.17**	3.37
*R* ^2^	.42				.44				.46			
*F* change	157.11**				5.35*				11.36**			

*p < .05. **p < .001.

### Depression

A multiple stepwise regression was conducted to examine which variables predicted the variance of depression. The variables of benefits (yes = 1 vs no = 0), long-term health conditions (yes = 1 vs no = 0), age (young adult [yes = 1, no = 0], middle-aged [yes = 1, no = 0], older adult [yes = 1, no = 0]), number of GP visits, number of hospital visits, exercise, use of public spaces, neighbourhood identification, strength of social network, loneliness and perceived risk of COVID-19 were inserted as predictors and depression was inserted as the dependent variable.

Loneliness was entered into Step 1 and explained 58% of the variance in depression. At Step 2, loneliness and exercise explained 59% of the variance in depression. At Step 3, loneliness, exercise and perceived risk of COVID-19 explained 61% of the variance in depression. The regression model was statistically significant [*F*(3, 477) = 112.489, *p* < .001; *R*^2^ = .611]. Of all predictors, loneliness was the most powerful, followed by perceived risk of COVID-19 and exercise.

### Anxiety

A multiple stepwise regression was conducted to examine which variables predicted the variance of anxiety. The variables of benefits (yes = 1 vs no = 0), age (young adult [yes = 1, no = 0], middle-aged [yes = 1, no = 0], older adult [yes = 1, no = 0]), number of GP visits, exercise, strength of social network, loneliness and perceived risk of COVID-19 were inserted as predictors, and anxiety was inserted as the dependent variable.

Loneliness was entered into Step 1 and explained 42% of the variance in anxiety. At Step 2, loneliness and being an older adult explained 44% of the variance in anxiety. At Step 3, loneliness, being an older adult and perceived risk of COVID-19 explained 46% of the variance in anxiety. The regression model was statistically significant [*F*(2, 213) = 61.722, *p* < .001; *R*^2^ = .461]. Of all predictors, loneliness was the most powerful, followed by perceived risk of COVID-19 and being an older adult.

The findings of the multiple regression analyses support hypothesis 5.

## Discussion

It is clear that people with long-term health conditions, those receiving benefits and those of lower income generally report lower neighbourhood identification, a less strong social network and engage in less exercise and less use of public spaces. They are, however, more likely to experience poor psychological outcomes, such as loneliness, depression and anxiety. This clarifies the established finding that income inequality is associated with poor psychological outcomes (Fone et al., 2007; Wildman, 2003). People with lower income are less likely to participate in their community perhaps because they possess less economic capital to do so, while those in receipt of benefits may face stigma and experience marginalisation, thereby limiting the amount of their community participation. Those receiving benefits also report poorer mental health. Similarly, having a long-term health condition appears to limit one’s social network, accentuate feelings of loneliness and to be associated with poor mental health outcomes. There were no significant differences in perceived risk of COVID-19 between these groups. However, those facing health and income inequalities are more marginalised and, thus, their capacity to cope with emerging stressors, such as perceived risk of COVID-19, may be limited.

Although it is often reported that greater age is associated with loneliness, anxiety and depression with the elderly being at especially high risk, this is not true of our sample of residents in Wandsworth. Older adults were in fact less likely to report loneliness, depression and anxiety than both younger and middle-aged adults. It is possible that older adults in our sample were less willing to acknowledge their loneliness, depression and anxiety since to do so may be an acknowledgement of weakness and, thus, contribute to an erosion of their independence (e.g. Rokach et al., 2004). A coping strategy on their part may be to attenuate or deny altogether these threatening aspects of their identity and experience (Breakwell, 2015). In partial support of hypothesis 1, middle-aged adults (aged 41–65) appear to be at highest risk of poor outcomes but they tend not to be the focus of research and intervention. They should be. It will be necessary to focus on addressing the disproportionate psychological and physical health impact that COVID-19 may be having on these vulnerable communities (i.e. middle-aged adults, those of lower income, those in receipt of benefits and those with long-term conditions) during the pandemic.

An especially important finding in this study is that neighbourhood identification – that is, feeling part of one’s local community, proud to be a member of it and connected to others in the community – is associated with a range of positive health outcomes. This was positively associated with both the amount of physical exercise undertaken and strength of social network. Consistent with the social cure perspective (Jetten et al., 2012), neighbourhood identification may act as a ‘gateway’ to a sense of community and indeed other group memberships, which can provide a sense of support, belonging and affiliation with others (Wakefield et al., 2019). Indeed, neighbourhood identification was also associated with having a stronger social network. This is consistent with research into place identity, which generally shows that place can come to form a central part of an individual’s identity, shaping cognition, affect and behaviour (Bonaiuto et al., 1996; Dixon et al., 2014). Indeed, place (in this context, Wandsworth) may constitute a fulcrum for neighbourhood identity, facilitating a sense of sharedness, commonality and belonging among residents. In view of recent research showing the benefits of a sense of community during the pandemic (e.g. Jetten et al., 2020), it would be advantageous to promote a stronger sense of neighbourhood identity among residents by emphasising the positive aspects of one’s neighbourhood, community involvement and interpersonal connectedness.

This study provides robust evidence that, when controlling for socio-economic and health inequalities, community participation, neighbourhood identification and strength of social network, the most important determinant of both depression and anxiety in this sample is loneliness. Although strength of social network (that is, the absence of social isolation) is associated with depression and anxiety, loneliness appears to explain poor mental health in our sample. This is an important finding because it clarifies that it is not necessarily the number of people that one knows, meets or speaks to, but rather the *subjective sense of being lonely* that affects people’s mental health. After all, it is quite possible for an individual to know, meet and speak with many individuals but for them still to experience a sense of loneliness (Tanskanen & Anttila, 2016). In addition to its impact on poor mental health, loneliness has been found to impede engagement with COVID-19 preventive measures, such as wearing a face mask (Stickley et al., 2020). It is therefore important that the subjective psychological construct of loneliness, in particular, is the focus of our efforts to improve people’s physical and psychological wellbeing. This will be especially relevant in the context of widespread social distancing measures to curb the spread of COVID-19.

Although the psychological burden of loneliness has been noted elsewhere (Matthews et al., 2016), our data suggest that it may be especially significant in the era of COVID-19, which may compound pre-existing psychological issues in the general population. Indeed, the multiple regression analyses indicated that perceived personal risk of COVID-19 was a significant correlate of poor mental health outcomes. Since COVID-19 constitutes a pervasive societal hazard which affects us all (Jaspal, Fino, et al., in press), perceived personal risk of infection appears to add significantly to predicting the variance of both depression and anxiety in the sample – over and above loneliness. As a novel stressor associated with the pandemic, the situational and involuntary perception of being at risk of COVID-19 may be stimulating anxious and depressive symptomatology, which will need to be managed effectively as resurgences of the disease are predicted and communicated to the general public under growing mistrust and uncertainty (Breakwell, 2020; Breakwell & Jaspal, in press).

In the multiple regression predicting depression, exercise was inversely associated with depression, suggesting that this may constitute a protective factor although it must be noted that the cross-sectional design of our study does not allow us to ascertain causality. The hypothesis of a causal role of exercise would be consistent with previous research showing the physiological and psychological benefits of exercise (Hassmén et al., 2000). There is likely to be a reciprocal relationship between loneliness and exercise in that people who engage in exercise (e.g. in group settings, that is with other people and with motivation from others) may feel less lonely. Furthermore, those who are lonely may be less inclined to engage in exercise. Regular exercise must continue to be encouraged in the general population, which may be challenging in view of renewed social distancing measures. Opportunities for, and guidance about, exercising regularly in a safe and socially distanced manner must be facilitated. Amid COVID-19 and the restrictions to curb its spread, we must ensure that people continue to feel connected to others (virtually and in a socially distanced manner) to decrease loneliness (Jetten et al., 2020) – the most important determinant of poor mental health in our study.

### Limitations and future directions

These findings must be considered with caution due to several limitations. First, only an online participant recruitment method was used, which means that this sample is not necessarily representative of the Wandsworth population. Indeed, the high prevalence of long-term health conditions reported in the sample was uncharacteristic of the population of Wandsworth. Moreover, men and ethnic minorities are under-represented in this sample. The sample, on average, was also highly educated. All of these factors mean that the most vulnerable sections of the Wandsworth population (e.g. those who do not engage with charities and community organisations, those with no access to the Internet, and those with low levels of literacy) may not have been reached during participant recruitment. Future research should continue to focus on those facing health and socio-economic inequalities using other sampling strategies to triangulate these findings. It is also noteworthy that, given the demographics of Wandsworth, the borough itself is not particularly representative of the UK population, suggesting that data from other towns and cities would be beneficial. Second, this cross-sectional study provides an empirical snapshot of mental health during the pandemic at one point in time, that is, when lockdown measures were being eased after the first outbreak. Cross-sequential research would be valuable as it would enable us to identify trends in mental health outcomes at different points during the pandemic, especially as resurgences of the virus and future national lockdowns are occurring. Third, the economic implications of COVID-19 and changes to one’s occupational identity (e.g. the need to work from home, job insecurity) have been major sources of uncertainty during the pandemic. It would be valuable to examine the impact of economic and job-related variables on mental health outcomes, especially in the commuter regions of London, where significant occupational changes have been imposed and experienced.
